# Predictors of Gestational Weight Gain in a Low-Income Hispanic Population: Sociodemographic Characteristics, Health Behaviors, and Psychosocial Stressors

**DOI:** 10.3390/ijerph17010352

**Published:** 2020-01-04

**Authors:** Cara D. Dolin, Rachel S. Gross, Andrea L. Deierlein, Lauren T. Berube, Michelle Katzow, Yasaman Yaghoubian, Sara G. Brubaker, Mary Jo Messito

**Affiliations:** 1 Department of Obstetrics and Gynecology, New York University School of Medicine, New York, NY 10016, USA; Yasaman.Yaghoubian@nyulangone.org (Y.Y.); Sara.Brubaker@nyulangone.org (S.G.B.); 2 Department of Obstetrics and Gynecology, University of Pennsylvania Perelman School of Medicine, Philadelphia, PA 19104, USA; 3 Department of Pediatrics, Bellevue Hospital Center, New York University School of Medicine, New York, NY 10016, USA; Rachel.Gross@nyulangone.org (R.S.G.); mkatzow@northwell.edu (M.K.); Mary.Messito@nyulangone.org (M.J.M.); 4 College of Global Public Health, New York University, New York, NY 10012, USA; ald8@nyu.edu (A.L.D.); lt1169@nyu.edu (L.T.B.)

**Keywords:** diet, gestational weight gain, Hispanic, physical activity, pregnancy, psychosocial stressors

## Abstract

Hispanic women have a higher prevalence of weight associated complications in pregnancy. This ethnic disparity is likely related to behavior patterns, social circumstances, environmental exposures, and access to healthcare, rather than biologic differences. The objective was to determine associations between sociodemographic characteristics, health behaviors, and psychosocial stressors and gestational weight gain (GWG) in low-income Hispanic women. During pregnancy, information on sociodemographic characteristics, health behaviors, and psychosocial stressors were collected. Linear regression estimated mean differences in GWG by selected predictors. Multinomial logistic regression estimated odds of inadequate and excessive GWG by selected predictors. Five-hundred and eight women were included, 38% had inadequate and 28% had excessive GWG; 57% with a normal pre-pregnancy BMI had inadequate GWG. Compared to women with normal BMI, women with overweight or obesity were more likely to have excessive GWG (aRRR = 1.88, 95% CI: 1.04, 3.40 and aRRR = 1.98, 95% CI: 1.08, 3.62, respectively). Mean total GWG was higher among women who were nulliparous (ß = 1.34 kg, 95% CI: 0.38, 2.29) and those who engaged in ≥3 h of screen time daily (ß = 0.98 kg, 95% CI: 0.02, 1.94), and lower among women who were physically active during pregnancy (ß = −1.00 kg, 95% CI: −1.99, −0.03). Eating breakfast daily was associated with lower risk of inadequate GWG (aRRR = 0.47, 95% CI: 0.26, 0.83). Depressive symptoms and poor adherence to dietary recommendations were prevalent, but none of the psychosocial or dietary variables were associated with GWG. In this cohort of primarily immigrant, low-income, Hispanic women, there were high rates of poor adherence to diet and physical activity recommendations, and a majority of women did not meet GWG guidelines. Modifiable health behaviors were associated with GWG, and their promotion should be included in prenatal care.

## 1. Introduction

Population-based studies have shown that less than one third of women in the United States gain the recommended amount of weight during pregnancy [1,2]. This has significant public health implications because both inadequate and excessive gestational weight gain (GWG) are associated with adverse maternal and neonatal outcomes [3]. Inadequate GWG is associated with low birthweight, preterm birth, and lower rates of breastfeeding, while excessive GWG is associated with postpartum weight retention, macrosomia, cesarean delivery, and long-term obesity in the child [3,4,5].

Hispanic women are the largest minority group in the United States (U.S.). The prevalence of obesity in Hispanic women is 50.6% [6]. Hispanic women are at increased risk for diet and weight associated pregnancy complications, such as gestational diabetes [7]. Evidence has demonstrated racial and ethnic disparities in obstetric outcomes, including higher prevalence of obesity and gestational diabetes among Hispanic women as compared to white women [8]. It is unlikely that biologic differences account for these disparities, but rather behavior, social circumstances, environmental exposures, and access to healthcare [8]. The contributions of specific sociodemographic characteristics, health behaviors, such as dietary intake and physical activity, and psychosocial stressors to GWG in this population are understudied. Therefore, the objective of this study was to determine the associations between sociodemographic characteristics, health behaviors, and psychosocial stressors and gestational weight gain in low-income Hispanic women.

## 2. Materials and Methods

### 2.1. Study Design and Sample

This study was a secondary analysis of data collected from pregnant women enrolled in the Starting Early Child Obesity Prevention Study, a randomized controlled trial aimed at reducing the incidence of childhood obesity. The full description of the study has been published previously [9]. Briefly, women receiving care at a large, public hospital were approached for trial enrollment between 28–32 weeks of pregnancy from October 2012–December 2014. Eligibility criteria included self-identifying as Hispanic, age ≥ 18 years old, fluent in English or Spanish, and having an uncomplicated singleton pregnancy without significant medical or psychiatric illness, homelessness, and/or fetal anomalies (*n* = 533). Informed consent was obtained from each participant. Women were included in the current analysis if they had completed baseline surveys, information on GWG, and known gestational age at delivery (*n* = 528). Women who were underweight prior to pregnancy (BMI < 18.5 kg/m^2^, *n* = 9) or missing information from baseline surveys (*n* = 11) were excluded. The final sample included 508 women (95.3%). This study was approved by the institutional review board of New York University School of Medicine, Albert Einstein College of Medicine, Bellevue Hospital Center, and New York City Health and Hospitals. It was registered on clinicaltrials.gov (NCT01541761).

### 2.2. Gestational Weight Gain

Maternal anthropometric measurements were obtained from electronic medical records. Pre-pregnancy body mass index (BMI, kg/m^2^) was calculated using measured height and weight at the initial prenatal clinic visit (mean gestational age in weeks (SD) = 7.8 (1.9)) and categorized as: Normal weight (BMI 18.5–24.9 kg/m^2^), overweight (BMI 25–29.9 kg/m^2^), and obese (BMI ≥ 30 kg/m^2^). If the initial prenatal clinic visit occurred at ≥12 weeks’ gestation (*n* = 125, 24.6%), then self-reported pre-pregnancy weight was used in calculations. To examine the use of the self-reported pre-pregnancy weights among women missing first measured weights, we compared the first measured weight and self-reported pre-pregnancy weight among women with both weights. The correlation coefficient between the two weights was very high (r = 0.98). GWG was determined by subtracting the measured weight at initial prenatal clinic visit (or self-reported pre-pregnancy weight) from the measured weight when the woman was admitted for delivery (mean gestational age in weeks (SD) = 39.4 (1.3)). Adequacy of GWG (inadequate, adequate, excessive) was determined for each study participant using the 2009 Institute of Medicine GWG recommendations, specific to pre-pregnancy BMI status (Table 1) [2].

### 2.3. Predictors of Gestational Weight Gain

Data on sociodemographic characteristics, health behaviors, and psychosocial stressors were collected through structured interviewer-administered surveys in English or Spanish by trained bilingual research staff during the third trimester of pregnancy.

### 2.4. Sociodemographic Characteristics

Women were asked about their parity, marital status, employment status, participation in the Special Supplemental Nutrition Assistance Program for Women, Infant and Children (WIC) and/or the Supplemental Nutrition Assistance Program (SNAP), primary language (Spanish or English), country of birth, and number of years spent living in the U.S.

### 2.5. Health Behaviors

Dietary intakes during the previous 12 months were collected using the Block 2005 Bilingual food frequency questionnaire [10]. Intakes of food groups were dichotomized as meeting daily recommendations according to the U.S. Department of Agriculture (USDA) Dietary Guidelines for Americans 2015–2020 [11]. Women were also asked about whether they consumed breakfast on a daily basis. Physical activity prior to and during pregnancy was assessed using questions taken from the 2011 Behavioral Risk Factor Surveillance System Physical Activity Rotating Core [12]. Total minutes of weekly moderate- or high-intensity physical activity were calculated and dichotomized as meeting the recommended level of at least 150 min of moderate activity per week [13]. Screen time was defined as the number of hours per day spent watching television or movies, playing video games, and/or using social media.

### 2.6. Psychosocial Stressors

Depressive symptoms during the past two weeks were assessed using the validated Patient Health Questionnaire-9 tool [14]. Household food insecurity was assessed using the USDA Core Food Security Module [15]. Continuous scores were generated from 10 questions (Cronbach α = 0.53) and dichotomized using recommended cut points. Women were classified as “food secure” if they reported no more than 2 food-insecure conditions and “food insecure” if they reported 3 or more. Financial difficulties and housing disrepair were measured using the Survey of Income and Program Participation [16]. Difficulties paying bills was assessed using the questions (1) “Have you had serious financial problems or been unable to pay monthly bills, rent, or mortgage during the past 12 months?”; (2) “Has there been a time when your household had service turned off by the gas or electric company, or the telephone company?” Continuous scores were generated on the basis of the sum of the responses (α = 0.53). A categorical variable was defined as a “yes” response to either of these questions. Housing disrepair was measured using the question “Are any of the following conditions present in your home?” Responses included (1) a leaking roof or ceiling, (2) a toilet, hot water heater, or other plumbing that does not work, (3) broken windows, (4) exposed electric wires, (5) rats, mice, roaches, or other insects, (6) holes in floor (large enough to trip in), and (7) open cracks or holes in the walls or ceiling. Continuous scores were generated on the basis of the number of housing conditions experienced (α = 0.51). A categorical variable for housing disrepair was defined as a “yes” response to any of the housing conditions. Neighborhood stress was assessed through questions from the Pregnancy Risk Assessment Monitoring System (PRAMS) [17]. Mothers were asked: “Did you do any of the following things because you felt it was unsafe to leave or return to the neighborhood where you live?”: (1) Miss doctor or other appointments, (2) limit grocery or other shopping, and (3) stay with other family members or friends. Responses were on the basis of a 5-point Likert scale (never, almost never, sometimes, fairly often, and always). Continuous scores were generated from the sum of the 3 questions (α = 0.59). A categorical variable was defined as never versus ever experiencing neighborhood stress.

### 2.7. Statistical Analysis

Data analysis was performed using Stata version 15.0 (College Station, TX, USA). Bivariable associations of categories of GWG adequacy and the selected maternal predictors, sociodemographic characteristics, health behaviors, and psychosocial stressors, were determined by chi-square tests or Fisher’s exact tests (for sample sizes < 5). Linear regression estimated mean differences in total GWG (kg), and multinomial logistic regression estimated relative risk ratios for categories of GWG adequacy (referent was adequate GWG) associated with maternal predictors (baseline models). Baseline models were adjusted for continuous pre-pregnancy BMI. All maternal predictors that were independently associated with total GWG or adequacy of GWG (*p* ≤ 0.05) in baseline models were included in final multivariable regression models.

## 3. Results

GWG outside of the recommended ranges were prevalent; 38% and 28% of women had inadequate and excessive GWG, respectively (Table 2). The majority of women were overweight or obese, <30 years old, married, and multiparous (Table 2). Most of the women reported Spanish as their preferred language (81%) and were born outside of the U.S. (81%), with approximately half being born in Mexico and the majority of others born in Ecuador, Dominican Republic, and Colombia. Among women who were born outside the U.S., 33% lived in the U.S. for at least ten years. Nearly all of the women reported receiving WIC benefits. Most women did not meet the recommended daily intakes of the selected dietary variables. Only 11% consumed ≥2.5 servings of vegetables, and only 14% met recommendations of ≥2 servings of whole fruit. Consumption of whole grains was particularly low, with only 5% meeting recommendations. Slightly more than half of the women reported being physically active prior to pregnancy, but only 32% met physical activity recommendations during pregnancy. One-third of the cohort engaged in ≥3 h of screen time daily. Psychosocial stressors of depressive symptoms, financial difficulties, housing disrepair, and food insecurity were prevalent, being reported by approximately 27–34% of the population (Table 2).

The distribution of GWG adequacy differed by maternal pre-pregnancy BMI status (*p* < 0.05); 57% of normal weight women had inadequate GWG, while 35% of overweight and 36% of obese women had excessive GWG (Table 2). In baseline multinomial logistic regression models (Table 3), pre-pregnancy normal weight, primary language of Spanish, being born in a Latin American country (not Mexico), and daily consumption of breakfast were associated with adequate GWG. In baseline linear regression analyses, mean total GWG was lower among women who met weekly physical activity recommendations. Mean total GWG was greater among women who were nulliparous or had ≥3 h of daily screen time. None of the dietary intake variables or psychosocial stressors were associated with total GWG or adequacy of GWG.

In multivariable multinomial regression models (Table 4), compared to women with normal BMI, women with pre-pregnancy overweight and obesity were more likely to have excessive GWG (aRRR: 1.88, 95% CI: 1.04, 3.40 and aRRR: 1.98; 95% CI: 1.08, 3.62, respectively). Consuming breakfast everyday was associated with a lower likelihood of inadequate GWG (aRRR: 0.47, 95% CI: 0.26, 0.83). In multivariable linear regression models, nulliparity, and engaging in ≥3 h of daily screen time were associated with approximately 1 kg greater total GWG (1.34 kg; 95% CI 0.38, 2.29 and 0.99 kg; 95% CI: 0.02, 1.94, respectively). Meeting physical activity recommendations of at least 150 min per week was associated with 1 kg lower total GWG (−1.00 kg; 95% CI −1.99, −0.03).

## 4. Discussion

In this study of urban, low-income, primarily immigrant Hispanic women, we identified several modifiable characteristics and health behaviors that may influence GWG. Greater odds of excessive GWG were observed among women who were overweight or obese prior to pregnancy. Daily breakfast consumption was associated with a decreased likelihood of inadequate GWG. Nulliparity and daily screen time ≥3 h were associated with greater total GWG, while meeting physical activity recommendations during pregnancy was associated with lower total GWG. Psychosocial stressors and specific dietary intakes were not predictive of total or adequacy of GWG.

A surprising observation in this study was that over one-third of women had inadequate GWG, of which half were normal weight prior to pregnancy. This prevalence of inadequate GWG is higher than what is commonly reported in the US, particularly among normal weight women. A large study utilizing data from over two million US birth certificates (2011–2012) found 17% of women had inadequate GWG and only 20% of those women had a normal prepregnancy BMI [18]. Discrepancies in prevalence estimates of GWG adequacy between studies may be partially due to differences in data collection methods of measured weights versus self-reported weights (often found on birth certificates). Self-reported pre-pregnancy weight, which is often under-reported, may lead to overestimation of GWG and underestimation in the prevalence of inadequate GWG [19,20]. Ethnicity may place a role in GWG. Similar to our findings, a study of women in Brazil found a 50.9% prevalence of inadequate GWG among women with a normal pre-pregnancy BMI [21]. In the US, a study using data from PRAMS found Hispanic ethnicity was associated with increased odds of inadequate GWG among normal weight women (OR 1.29; 95% CI 1.07–1.56) (1); however, among predominantly Puerto Rican women living in the US, the prevalence of inadequate GWG among those with normal pre-pregnancy BMI (21.9%) was comparable to national estimates for all women [22]. The higher prevalence of inadequate GWG in the current study suggests that GWG in women of Hispanic ethnicity may differ based on country of origin. Other studies have found that, among immigrants, level of acculturation plays a role in GWG, with less acculturated women receiving advice regarding weight gain from family rather than healthcare providers [23,24].

In this pregnant population, most women consumed below the recommended number of servings of major nutritious food groups. Similar findings have been reported among non-pregnant Hispanic women participating in SNAP [25]. We found no associations between meeting dietary recommendations for selected food groups and GWG. Evidence supporting the influence of diet on GWG is inconsistent and likely dependent on the methods used to assess and analyze dietary data [26]. We found that consuming breakfast was associated with lower odds of inadequate GWG. While the contribution of breakfast to GWG has not been previously studied, skipping breakfast is associated with poor diet quality, obesity, and cardiovascular risk in non-pregnant adult populations [27,28,29]. A nutrient-dense diet during pregnancy is vital for optimal growth and development of the fetus. Diet is also the first-line treatment for gestational diabetes, a condition that is prevalent among Hispanic women [7]. Interventions and public health campaigns targeting low-income Hispanic women, both during preconception and pregnancy, should focus on nutrition education and helping women achieve dietary recommendations.

Physical activity during pregnancy was associated with ~1 kg decrease in total GWG, which is consistent with findings from randomized trials of exercise to prevent excessive GWG [30]. Similar to our results, other studies have not found an association between physical activity and adequacy of GWG in low-income populations [31,32]. Physical activity during pregnancy not only helps maintain cardiovascular fitness during pregnancy, but may also decrease the risk of complications in pregnancy and during delivery [1,30]. In the current study, approximately half of women prior to pregnancy and two-thirds of women during pregnancy did not meet physical activity recommendations. Promoting physical activity and decreasing sedentary behaviors may be important modifiable targets to address during prenatal care. This highlights the need to focus interventions on educating about misconceptions about exercise in pregnancy and encouraging women, especially low-income Hispanic women, to stay active during pregnancy.

None of the selected psychosocial stressors were associated with GWG. Our findings are consistent with other literature that has failed to demonstrate an association between excessive GWG and WIC enrollment, pre-pregnancy depression, or partner abuse. In one study, high rates of perceived stress were associated with both inadequate and excessive GWG in women with a normal BMI, but there was no association between stress and GWG in women with overweight or obesity [33]. The relationship between individual psychosocial stressors and GWG is likely complex. Interestingly, there was a high prevalence (33.2%) of depressive symptoms in this population of primarily Hispanic immigrants. Systematic reviews have found the prevalence of perinatal depression to be about 11% [34,35]. This finding highlights the importance of antenatal screening for depression, specifically in Hispanic women, a group at higher risk of perinatal depression [34,36].

There are several limitations of this study. The majority of our data was based on interviews, which were subject to social desirability bias. In addition, the dietary and exercise information was collected at only one time point in the third trimester, and may be subject to recall bias. Additionally, there may be other factors contributing to GWG that were not captured in our analysis. This study also had several strengths. Most participants’ GWG information was obtained through measured weights rather than participant self-report. The survey questions used in this study were adapted from validated nationally used questionnaires. The study population targeted low-income, Hispanic immigrants living in the US. The needs of this underserved community are important to consider both on an individual scale, as patients in the clinical care setting, and on a public health scale, as interventions are designed to improve short and long-term maternal and child health outcomes [37].

## 5. Conclusions

In a population of primarily immigrant, low-income Hispanic women, we found high rates of overweight and obesity, poor adherence to diet and physical activity recommendations, and a majority of women not meeting GWG guidelines. Since less exercise and greater screen time were associated with higher GWG and daily breakfast was protective against inadequate GWG, promotion of these factors should be included as part of prenatal care. The relatively high rate of inadequate GWG among women with pre-pregnancy BMI in the normal range suggests a need for long-term follow up of these women and infants to understand whether current GWG guidelines adequately predict long-term maternal-child outcomes for this group. The high risk of excess GWG associated with pre-pregnancy overweight and obesity points to the need for more intensive prevention programs for these women to avoid the increased risk of perinatal complications and the intergenerational transmission of obesity to their children.

## Figures and Tables

**Table 1:** Institute of Medicine gestational weight gain recommendations and mean and range of gestational weight gain among women participating in the Starting Early Obesity Prevention Study, stratified by pre-pregnancy body mass index.

Pre-Pregnancy BMI	IOM GWG Recommendations (kg)	Starting Early Obesity Prevention Study	
		Mean GWG (kg ± SD)	Range (kg)
Normal 18.5–24.9 kg/m^2^	11.3–15.9	11.1 ± 4.8	−3.5–32.4
Overweight 25–29.9 kg/m^2^	6.8–11.3	10.3 ± 5.4	−0.4–31.5
Obese ≥30.0 kg/m^2^	5.0–9.1	8.1 ± 5.4	−3.8–25.5

IOM, Institute of Medicine; GWG, gestational weight gain; BMI, Body Mass Index.

**Table 2:** Distributions of sociodemographic characteristics, health behaviors, and psychosocial stressors among low-income Hispanic women participating in the Starting Early Obesity Prevention Study, stratified by gestational weight gain adequacy (*n* = 508).

Characteristic	All Women	Inadequate GWG (*n* = 194)	Adequate GWG (*n* = 182)	Excessive GWG (*n* = 143)
	*n* (%)	*n* (%)	*n* (%)	*n* (%)
**Sociodemographic Characteristics**				
Prepregnancy BMI				
Normal	176 (35)	100 (57)	52 (30)	24 (14) ***
Overweight	172 (34)	45 (26)	67 (39)	60 (35)
Obese	160 (32)	45 (28)	58 (36)	57 (36)
Age (years)				
<25	178 (35)	74 (42)	59 (33)	45 (25)
25 < 30	142 (28)	50 (35)	47 (33)	45 (32)
30 < 35	105 (21)	40 (38)	36 (34)	29 (28)
≥35	83 (16)	26 (31)	35 (42)	22 (27)
Primary language Spanish				
No	97 (19)	42 (43)	25 (26)	30 (31)
Yes	411 (81)	148 (36)	152 (37)	111 (27)
Country of Birth				
United States	98 (19)	42 (43)	26 (27)	30 (31)
Mexico	243 (48)	93 (38)	86 (35)	64 (26)
Other Latin Countries	167 (33)	55 (33)	65 (39)	47 (28)
Years living in United States				
≤5	106 (21)	36 (34)	40 (38)	30 (28)
>5–10	134 (26)	48 (36)	55 (41)	31 (23)
>10–20	144 (28)	56 (39)	45 (31)	43 (30)
>20 years or U.S. born	124 (24)	50 (40)	37 (30)	37 (30)
Nulliparous				
No	320 (63)	126 (39)	109 (34)	85 (27)
Yes	188 (37)	64 (34)	68 (36)	56 (30)
Married/Living with partner				
No	141 (28)	54 (38)	48 (34)	39 (28)
Yes	367 (72)	136 (37)	129 (35)	102 (28)
Currently employed				
No	377 (74)	146 (39)	129 (34)	102 (27)
Yes	131 (26)	44 (34)	48 (37)	39 (30)
Received WIC				
No	64 (13)	28 (44)	17 (27)	19 (27)
Yes	444 (87)	162 (36)	160 (36)	122 (27)
Received SNAP				
No	325 (64)	119 (36)	113 (35)	93 (29)
Yes	183 (36)	71 (39)	64 (35)	48 (26)
**Health Behaviors**				
Dairy, ≥3 servings/day				
No	388 (76)	145 (37)	131 (34)	112 (29)
Yes	120 (24)	45 (38)	46 (38)	29 (24)
Vegetables, ≥2.5 servings/day				
No	454 (89)	171 (38)	159 (35)	124 (27)
Yes	54 (11)	35 (19)	33 (18)	17 (31)
Whole Fruit, ≥2 servings/day				
No	436 (86)	167 (38)	151 (35)	118 (27)
Yes	72 (14)	23 (32)	26 (36)	23 (32)
Whole Grains, ≥3 servings/day				
No	485 (95)	187 (39)	165 (34)	133 (27) *
Yes	23 (5)	3 (13)	12 (52)	8 (35)
Refined Grains, ≤3 servings/day				
No	346 (68)	131 (38)	125 (36)	90 (26)
Yes	162 (32)	59 (36)	52 (32)	51 (31)
Protein, ≥5.5 servings/day				
No	366 (72)	131 (36)	130 (36)	105 (29)
Yes	142 (28)	59 (42)	47 (33)	36 (25)
Eats breakfast every day				
No	94 (19)	42 (45)	24 (25)	28 (30)
Yes	414 (81)	148 (36)	153 (37)	113 (27)
Met physical activity recommendations prior to pregnancy				
No	230 (45)	86 (37)	82 (36)	62 (27)
Yes	278 (55)	104 (37)	95 (34)	79 (28)
Met physical activity recommendations during pregnancy				
No	344 (68)	128 (37)	117 (34)	99 (29)
Yes	164 (32)	62 (38)	60 (37)	42 (26)
≥3 h screen time/day				
No	340 (67)	135 (40)	118 (35)	87 (26)
Yes	168 (33)	55 (33)	59 (35)	54 (32)
**Psychosocial Stressors**				
Depressive symptoms				
No	342 (67)	123 (36)	118 (35)	101 (30)
Yes	166 (33)	67 (40)	59 (36)	40 (24)
Financial Difficulties				
No	372 (73)	137 (37)	132 (35)	103 (28)
Yes	136 (27)	53 (39)	45 (33)	38 (28)
Housing Disrepair				
No	333 (66)	123 (37)	111 (33)	100 (30)
Yes	175 (34)	67 (38)	67 (38)	41 (23)
Food Insecurity				
No	350 (69)	127 (36)	125 (36)	98 (28)
Yes	158 (31)	63 (40)	52 (33)	43 (27)
Neighborhood Stress				
No	465 (92)	172 (37)	164 (35)	129 (28)
Yes	43 (8)	18 (40)	13 (31)	12 (29)

GWG, gestational weight gain; BMI, body mass index; SNAP, Supplemental Nutrition Assistance Program; WIC, Women, Infant and Children. *p* values are from chi-square test or Fisher’s exact test, * *p *< 0.05, *** *p* < 0.0001.

**Table 3:** Multinomial logistic regression and linear regression estimating associations of gestational weight gain with sociodemographic characteristics, health behaviors, and psychosocial stressors (*n* = 508).

Characteristic	Inadequate GWG		Excessive GWG		Total GWG	
	RRR	95% CI	RRR	95% CI	kg	95% CI
**Sociodemographic Characteristics**						
Pre-pregnancy BMI						
Normal Weight	Ref		Ref		Ref	
Overweight	0.35	0.21, 0.58 ***	1.94	1.07, 3.52 *	−0.82	−1.93, 0.28
Obese	0.40	0.24, 0.67 **	2.13	1.16, 3.90 *	−3.02	−4.14, −1.90 ***
Age (years)						
<25	Ref		Ref		Ref	
25 < 30	0.89	0.53, 1.51	1.23	0.70, 2.16	0.27	−0.88, 1.42
30 < 35	0.98	0.55, 1.74	1.00	0.53, 1.89	−0.49	−1.76, 0.78
≥35	0.65	0.35, 1.20	0.80	0.41, 1.54	−0.84	−2.20, 0.52
Primary language Spanish	0.58	0.34, 1.00 *	0.62	0.34, 1.11	−0.33	−1.48, 0.82
Country of Birth						
United States	Ref		Ref		Ref	
Mexico	0.65	0.37, 1.16	0.66	0.36, 1.23	−0.96	−2.17, 0.26
Other Latin Countries	0.49	0.27, 0.91 *	0.65	0.34, 1.26	0.01	−1.29, 1.30
Years Living in U.S.						
>20 years or U.S. born	Ref		Ref		Ref	
≤5	0.60	0.32, 1.12	0.79	0.40, 1.54	0.47	−0.89, 1.83
>5–10	0.61	0.34, 1.09	0.58	0.31, 1.11	−0.67	−1.95, 0.60
>10–20	0.92	0.51, 1.65	0.97	0.52, 1.80	−0.65	−1.89, 0.60
Nulliparous	0.73	0.47, 1.12	1.12	0.70, 1.78	1.23	0.28, 2.18 **
Married/Living with partner	0.95	0.60, 1.51	0.97	0.59, 1.59	−0.56	−1.57, 0.45
Currently Employed	0.83	0.52, 1.34	1.01	0.61, 1.66	0.06	−0.97, 1.10
WIC	0.64	0.34, 1.22	0.66	0.33, 1.34	0.50	−0.86, 1.87
SNAP	1.10	0.71, 1.68	0.90	0.56, 1.43	−0.17	−1.11, 0.77
**Health Behaviors**						
Dairy, ≥3 servings/day	0.85	0.53, 1.38	0.75	0.44, 1.27	−0.73	−1.79, 0.33
Vegetables, ≥2.5 servings/day	0.98	0.49, 1.94	1.20	0.59, 2.43	0.07	−1.40, 1.53
Whole Fruit, ≥2 servings/day	0.74	0.40, 1.36	1.19	0.64, 2.20	0.19	−1.11, 1.50
Refined Grains, ≤3 servings/day	1.11	0.71, 1.74	1.35	0.84, 2.16	0.55	−0.42, 1.52
Protein, ≥5.5 servings/day	1.21	0.76, 1.91	0.96	0.58, 1.59	−0.41	−1.42, 0.59
Eats breakfast every day	0.49	0.28, 0.86 *	0.66	0.36, 1.22	0.06	−1.12, 1.23
Met PA Recommendations prior to pregnancy	1.02	0.68, 1.55	1.11	0.71, 1.74	−0.13	−1.04, 0.78
Met PA Recommendations during pregnancy	1.00	0.64, 1.55	0.80	0.49, 1.29	−0.97	−1.94, −0.01 *
≥3 h screen time/day	0.80	0.51, 1.25	1.25	0.79, 1.99	1.04	0.08, 2.00 *
**Psychosocial Stressors**						
Depressive symptoms	1.06	0.69, 1.63	0.80	0.50, 1.30	−0.60	−1.57, 0.36
Financial Difficulties	1.17	0.73, 1.87	1.07	0.65, 1.77	0.01	−1.00, 1.03
Housing Disrepair	0.90	0.59, 1.37	0.67	0.42, 1.08	−0.84	−1.78, 0.11
Food Insecurity	1.18	0.76, 1.84	1.06	0.65, 1.72	0.24	−0.73, 1.22
Neighborhood Stress	1.30	0.61, 2.74	1.18	0.52, 2.68	−0.22	−1.85, 1.40

RRR, relative risk ratio; CI, confidence interval; GWG, gestational weight gain; BMI, body mass index; SNAP, Supplemental Nutrition Assistance Program; WIC, Women, Infant and Children; PA, physical activity. For multinomial regression, reference is adequate GWG. All models are adjusted for pre-pregnancy BMI. Model for Whole grains, ≥3 servings/day omitted due to small numbers (*n* < 5). * *p* values are indicated with asterisks, with * *p* < 0.05, ** *p* < 0.01, *** *p* < 0.001.

**Table 4:** Multivariable multinomial logistic regression and multivariable linear regression estimating associations of gestational weight gain with sociodemographic characteristics and health behaviors (*n* = 508).

Characteristic	Inadequate GWG		Excessive GWG		Total GWG (kg)	
	aRRR	95% CI	aRRR	95% CI	kg	95% CI
Pre-pregnancy BMI						
Normal Weight	Ref		Ref		Ref	
Overweight	0.34	0.20, 0.57 ***	1.88	1.04, 3.40 *	−0.66	−1.75, 0.44
Obese	0.36	0.22, 0.62 ***	1.98	1.08, 3.62 *	−2.63	−3.76, −1.49 **
Primary language Spanish	0.77	0.35, 1.69	0.57	0.24, 1.35		
Country of Birth						
United States	Ref		Ref			
Mexico	1.01	0.46, 2.24	1.04	0.43, 2.47		
Other Latin Countries	0.73	0.32, 1.67	1.06	0.44, 2.59		
Nulliparous					1.34	0.38, 2.29 *
Eats breakfast every day	0.47	0.26, 0.83 *	0.65	0.35, 1.18		
Met physical activity recommendations during pregnancy					−1.00	−1.99, −0.03 *
Engaged in ≥3 h screen time/day					0.98	0.02, 1.94

aRRR, adjusted relative risk ratio; CI, confidence interval; GWG, gestational weight gain; BMI, body mass. index: * *p* values are indicated with asterisks, with * *p* < 0.05, ** *p* < 0.001.

## References

[1] Deputy N.P., Sharma A.J., Kim S.Y., Hinkle S.N. (2015). Prevalence and characteristics associated with gestational weight gain adequacy. Obs. Gynecol..

[2] IOM, NRC (2009). Weight Gain During Pregnancy:Reexamining the Guidelines.

[3] Kominiarek M.A., Peaceman A.M. (2017). Gestational weight gain. Am. J. Obstet. Gynecol..

[4] Goldstein R.F., Abell S.K., Ranasinha S., Misso M., Boyle J.A., Black M.H., Li N., Hu G., Corrado F., Rode L. (2017). Association of Gestational Weight Gain With Maternal and Infant Outcomes: A Systematic Review and Meta-analysis. Jama.

[5] Voerman E., Santos S., Inskip H., Amiano P., Barros H., Charles M.A., Chatzi L., Chrousos G.P., Corpeleijn E., Crozier S. (2019). Association of Gestational Weight Gain With Adverse Maternal and Infant Outcomes. Jama.

[6] Hales C.M., Carroll M.D., Fryar C.D., Ogden C.L. (2017). Prevalence of Obesity Among Adults and Youth: United States, 2015–2016. NCHS Data Brief.

[7] Caughey A.B., Cheng Y.W., Stotland N.E., Washington A.E., Escobar G.J. (2010). Maternal and paternal race/ethnicity are both associated with gestational diabetes. Am. J. Obstet. Gynecol..

[8] Bryant A.S., Worjoloh A., Caughey A.B., Washington A.E. (2010). Racial/ethnic disparities in obstetric outcomes and care: Prevalence and determinants. Am. J. Obstet. Gynecol..

[9] Gross R.S., Mendelsohn A.L., Gross M.B., Scheinmann R., Messito M.J. (2016). Randomized Controlled Trial of a Primary Care-Based Child Obesity Prevention Intervention on Infant Feeding Practices. J. Pediatr..

[10] Block G., Wakimoto P., Jensen C., Mandel S., Green R.R. (2006). Validation of a food frequency questionnaire for Hispanics. Prev. Chronic Dis..

[11] USDA (2015). Dietary Guidelines for Americans 2015–2020.

[12] CDC (2011). A Data Users Guide to the BRFSS Physical Activity Questions.

[13] ACOG (2015). Committee Opinion No. 650: Physical Activity and Exercise During Pregnancy and the Postpartum Period. Obstet. Gynecol..

[14] Kroenke K., Spitzer R.L., Williams J.B. (2001). The PHQ-9: Validity of a brief depression severity measure. J. Gen. Intern. Med..

[15] Bickel G., Nord M., Price C., Hamilton W., Cook J. (2000). Guide to Measuring Household Food Security, Revised 2000.

[16] Baehr A., Pena J.C., Hu D.J. (2015). Racial and Ethnic Disparities in Adverse Drug Events: A Systematic Review of the Literature. J. Racial Ethn. Health Disparities.

[17] Shulman H.B., Gilbert B.C., Msphbrenda C.G., Lansky A. (2006). The Pregnancy Risk Assessment Monitoring System (PRAMS): Current methods and evaluation of 2001 response rates. Public Health Rep. (Washington, DC: 1974).

[18] Truong Y.N., Yee L.M., Caughey A.B., Cheng Y.W. (2015). Weight gain in pregnancy: Does the Institute of Medicine have it right?. Am. J. Obstet. Gynecol..

[19] Wright C.S., Weiner M., Localio R., Song L., Chen P., Rubin D. (2012). Misreport of gestational weight gain (GWG) in birth certificate data. Matern. Child Health J..

[20] Bodnar L.M., Abrams B., Bertolet M., Gernand A.D., Parisi S.M., Himes K.P., Lash T.L. (2014). Validity of birth certificate-derived maternal weight data. Paediatr. Perinat. Epidemiol..

[21] Rodrigues P.L., De Oliveira L.C., Brito Ados S., Kac G. (2010). Determinant factors of insufficient and excessive gestational weight gain and maternal-child adverse outcomes. Nutr. (Burbank Los Angeles Cty. Calif.).

[22] Chasan-Taber L., Schmidt M.D., Pekow P., Sternfeld B., Solomon C.G., Markenson G. (2008). Predictors of excessive and inadequate gestational weight gain in Hispanic women. Obes. (Silver SpringMd).

[23] Tovar A., Chasan-Taber L., Bermudez O.I., Hyatt R.R., Must A. (2010). Knowledge, attitudes, and beliefs regarding weight gain during pregnancy among Hispanic women. Matern. Child Health J..

[24] Fletcher G.E., Teeters L., Schlundt D., Bonnet K., Heerman W.J. (2018). Maternal conception of gestational weight gain among Latinas: A qualitative study. Health Psychol..

[25] Jun S., Thuppal S.V. (2018). Poor Dietary Guidelines Compliance among Low-Income Women Eligible for Supplemental Nutrition Assistance Program-Education (SNAP-Ed). Nutrients.

[26] Streuling I., Beyerlein A., Rosenfeld E., Schukat B., von Kries R. (2011). Weight gain and dietary intake during pregnancy in industrialized countries—A systematic review of observational studies. J. Perinat. Med..

[27] Nicklas T.A., Myers L., Reger C., Beech B., Berenson G.S. (1998). Impact of breakfast consumption on nutritional adequacy of the diets of young adults in Bogalusa, Louisiana: Ethnic and gender contrasts. J. Am. Diet. Assoc..

[28] Deshmukh-Taskar P.R., Radcliffe J.D., Liu Y., Nicklas T.A. (2010). Do breakfast skipping and breakfast type affect energy intake, nutrient intake, nutrient adequacy, and diet quality in young adults? NHANES 1999–2002. J. Am. Coll. Nutr..

[29] Deshmukh-Taskar P., Nicklas T.A., Radcliffe J.D., O'Neil C.E., Liu Y. (2013). The relationship of breakfast skipping and type of breakfast consumed with overweight/obesity, abdominal obesity, other cardiometabolic risk factors and the metabolic syndrome in young adults. The National Health and Nutrition Examination Survey (NHANES): 1999–2006. Public Health Nutr..

[30] Muktabhant B., Lawrie T.A., Lumbiganon P., Laopaiboon M. (2015). Diet or exercise, or both, for preventing excessive weight gain in pregnancy. Cochrane Database Syst. Rev..

[31] Chasan-Taber L., Silveira M., Lynch K.E., Pekow P., Solomon C.G., Markenson G. (2014). Physical activity and gestational weight gain in Hispanic women. Obes. (Silver SpringMd).

[32] Herring S.J., Nelson D.B., Davey A., Klotz A.A., Oken E., Foster G.D. (2012). Determinants of excessive gestational weight gain in urban, low-income women. Women’s Health Issues.

[33] Kubo A., Ferrara A., Brown S.D., Ehrlich S.F., Tsai A.L., Quesenberry C.P., Crites Y., Hedderson M.M. (2017). Perceived psychosocial stress and gestational weight gain among women with gestational diabetes. PLoS ONE.

[34] Woody C.A., Ferrari A.J., Siskind D.J., Whiteford H.A., Harris M.G. (2017). A systematic review and meta-regression of the prevalence and incidence of perinatal depression. J. Affect. Disord..

[35] Gavin N.I., Gaynes B.N., Lohr K.N., Meltzer-Brody S., Gartlehner G., Swinson T. (2005). Perinatal depression: A systematic review of prevalence and incidence. Obs. Gynecol..

[36] Lavizzo-Mourey R. (2015). Use financial incentives to reduce racial and ethnic disparities in healthcare. Mod. Healthc..

[37] ACOG (2018). Committee Opinion No. 729: Importance of Social Determinants of Health and Cultural Awareness in the Delivery of Reproductive Health Care. Obs. Gynecol..

